# Coupled Fe_3_O_4_‐Cluster Precipitates and Single Fe‐Atom Catalysts Can Boost Oxygen Reduction via Concomitantly Accelerated Water Dissociation

**DOI:** 10.1002/anie.5493485

**Published:** 2026-07-09

**Authors:** Cheng‐Kai Du, Xiongyi Liang, Fei‐Xiang Ma, Yutong Li, Zheng‐Qi Liu, Long Ma, Zeng Li, Liang Zhen, Yan Huang, Xiao Cheng Zeng, Cheng‐Yan Xu

**Affiliations:** ^1^ Sauvage Laboratory for Smart Materials School of Materials Science and Engineering Harbin Institute of Technology (Shenzhen) Shenzhen China; ^2^ Department of Materials Science and Engineering City University of Hong Kong Kowloon Hong Kong China; ^3^ Department of Chemistry City University of Hong Kong Kowloon Hong Kong China; ^4^ Shenzhen Research Institute City University of Hong Kong Shenzhen China

**Keywords:** ab initio calculations, Fe_3_O_4_‐cluster precipitates, oxygen reduction reaction, single‐atom catalysts, water dissociation

## Abstract

Fe–N–C single‐atom catalysts (SACs) can deliver high activity for catalyzing the oxygen reduction reaction (ORR) in alkaline conditions. However, the sluggish water dissociation upon Fe–N_4_ active sites limits their proton‐coupled electron transfer (PCET) capability, impeding their practical applications like anion‐exchange membrane fuel cells (AEMFCs). Here, inspired by the known nanoprecipitation behavior in solid‐solution alloys, a residual‐oxygen‐assisted precipitation strategy is undertaken to fabricate coupled Fe_3_O_4_‐cluster precipitates along with single Fe‐atom catalysts (Fe_3_O_4_/Fe_SA_@NC), where Fe_3_O_4_ clusters are generated by slight oxidation and local enrichment of Fe atoms in the Fe_SA_@NC matrix. *Operando* spectroscopy and theoretical calculations suggest that the Fe_3_O_4_‐cluster precipitates not only induce asymmetric electronic structures of the Fe‐N_4_ active center to optimize the OH* adsorption, but also accelerate the water dissociation on Fe‐N_4_ sites to boost the PCET steps, thereby promoting the ORR. Notably, the coupled Fe_3_O_4_/Fe_SA_@NC exhibits superb alkaline ORR performance with a high half‐wave potential of 0.953 V versus RHE. When employed as cathode catalysts, the Fe_3_O_4_/Fe_SA_@NC demonstrates a high peak power density of 909.3 mW cm^−2^ and 219.4 mW cm^−2^ in AEMFCs and Zn‐air batteries, respectively, far exceeding that of the commercial Pt/C catalyst. The novel coupled metal‐oxide cluster/SAC strategy can be exploited as a generic approach for improving electrocatalytic performance.

## Graphical abstract

Fe_3_O_4_ clusters can be precipitated onto Fe–N–C SACs through a residual‐oxygen‐assisted precipitation approach. Compelling evidence, including *operando* spectroscopy and ab initio calculations, suggests that Fe_3_O_4_‐cluster precipitates not only induce asymmetric electronic structures of Fe‐N_4_ active center to optimize the OH* adsorption, but also accelerate the water dissociation on Fe‐N_4_ sites to boost the PCET steps, thereby promoting the ORR.

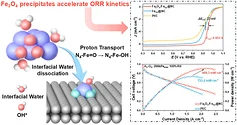

## 1 Introduction

Anion exchange membrane fuel cells (AEMFCs) and metal‐air batteries represent advanced sustainable electrochemical technologies to relieve the forthcoming energy crisis [1, 2, 3]. However, the sluggish oxygen reduction reactions (ORR) kinetics arising from the complex four‐electron reaction pathway  limits the performance of these devices [4, 5]. Currently, ORR catalysts still rely heavily on the costly and limited platinum‐group metals (PGMs), unavoidably hindering the large‐scale deployment of the technologies. Consequently, substantial efforts have been devoted to exploring PGM‐free alternatives for ORRs [6, 7]. Among the diverse candidates, Fe–N–C single‐atom catalysts (SACs) featuring atomic Fe–N_4_ sites stand out as the most promising catalysts for ORR, owing to their exceptional intrinsic activities, well‐defined active sites, and high stabilities [8, 9, 10]. Generally, the four‐electron ORR in alkaline conditions entails a sequence of two key proton‐coupled electron transfer (PCET) steps, namely *O_2_ → *OOH and *O → *OH, in which the protons are provided by water dissociation. Fe–N–C SACs often deliver strong O_2_ adsorption and O═O bond cleavage during the ORR process [11, 12]. However, the adsorption energy of PCET‐associated oxygen intermediates (*OOH and *OH) on Fe–N_4_ sites remains a major limitation. Therefore, fine‐tuning the electronic structure of Fe–N_4_ sites is crucial to lowering energy barriers of PCET‐associated ORR intermediates, thus ultimately enhancing the performance of Fe–N–C SACs [13, 14].

To enhance the intrinsic activity of Fe–N–C SACs, many strategies have been developed, such as engineering adjacent metal atoms and introducing axial ligands (e.g., OH, Cl, F) to the metal active centers [15, 16, 17, 18]. Recently, Fe–N–C SACs modified with multinuclear clusters have emerged as a promising approach for enhancing the ORR activities and stabilities in alkaline media [19, 20]. For instance, the Fe_2_N cluster liganded Fe–N_4_ structure can lower the *OH desorption energy barrier, thereby significantly enhancing the ORR activity of Fe–N_4_ sites [21]. Nevertheless, most research efforts have been devoted to the electronic structure regulation of Fe–N_4_ sites, while the influence of multinuclear clusters on PCET‐coupled ORR intermediates on Fe–N_4_ sites is still less known [22, 23]. A deeper mechanistic understanding of the synergy between single‐atom sites and multinuclear clusters in PCET coupled ORR steps, along with developing reliable synthetic methods to produce cluster‐SAC hybrid catalysts, is highly desirable. On account of the classic nanoprecipitation behavior in solid‐solution alloys [24, 25, 26], partial metal atoms in SACs can precipitate to form clusters under specific conditions, thereby forming a unique coupled cluster‐SAC structure. Such in situ nanoprecipitation can assure intimate contact between the clusters and surrounding metal atoms, thus offering high potential for boosting the PCET‐coupled steps during the ORR [27].

Herein, using a pre‐synthesized single‐atom Fe‐N‐C material as a model, Fe_3_O_4_ clusters were precipitated in situ within the Fe–N–C SAC through a residual‐oxygen‐assisted precipitation approach, forming a well‐defined coupled Fe_3_O_4_‐cluster/Fe–N_4_ structure (denoted as Fe_3_O_4_/Fe_SA_@NC). In Fe_3_O_4_/Fe_SA_@NC, the nearby Fe_3_O_4_‐cluster precipitates are proposed to promote interfacial water dissociation, thereby strengthening hydrogen‐bonding interactions with oxygen intermediates and correlating with reduced kinetic barriers for the PCET steps, which can be supported by in situ attenuated total reflection surface‐enhanced infrared absorption spectroscopy (ATR‐SEIRAS) and theoretical calculations. The Fe_3_O_4_/Fe_SA_@NC catalyst yields a highly positive half‐wave potential (E_1/2_) of 0.953 V in alkaline media, along with robust performance, including high peak power densities and durability in both AEMFC and Zn‐air batteries (ZABs). This work offers a promising strategy for in situ synthesis of metal oxide clusters on SACs and provides new insights into the role of interfacial water in PCET‐involved electrocatalysis.

## 2 Results and Discussion

### 2.1 Design, Synthesis, and Characterizations of Fe_3_O_4_/Fe_SA_@NC

**FIGURE 1:**
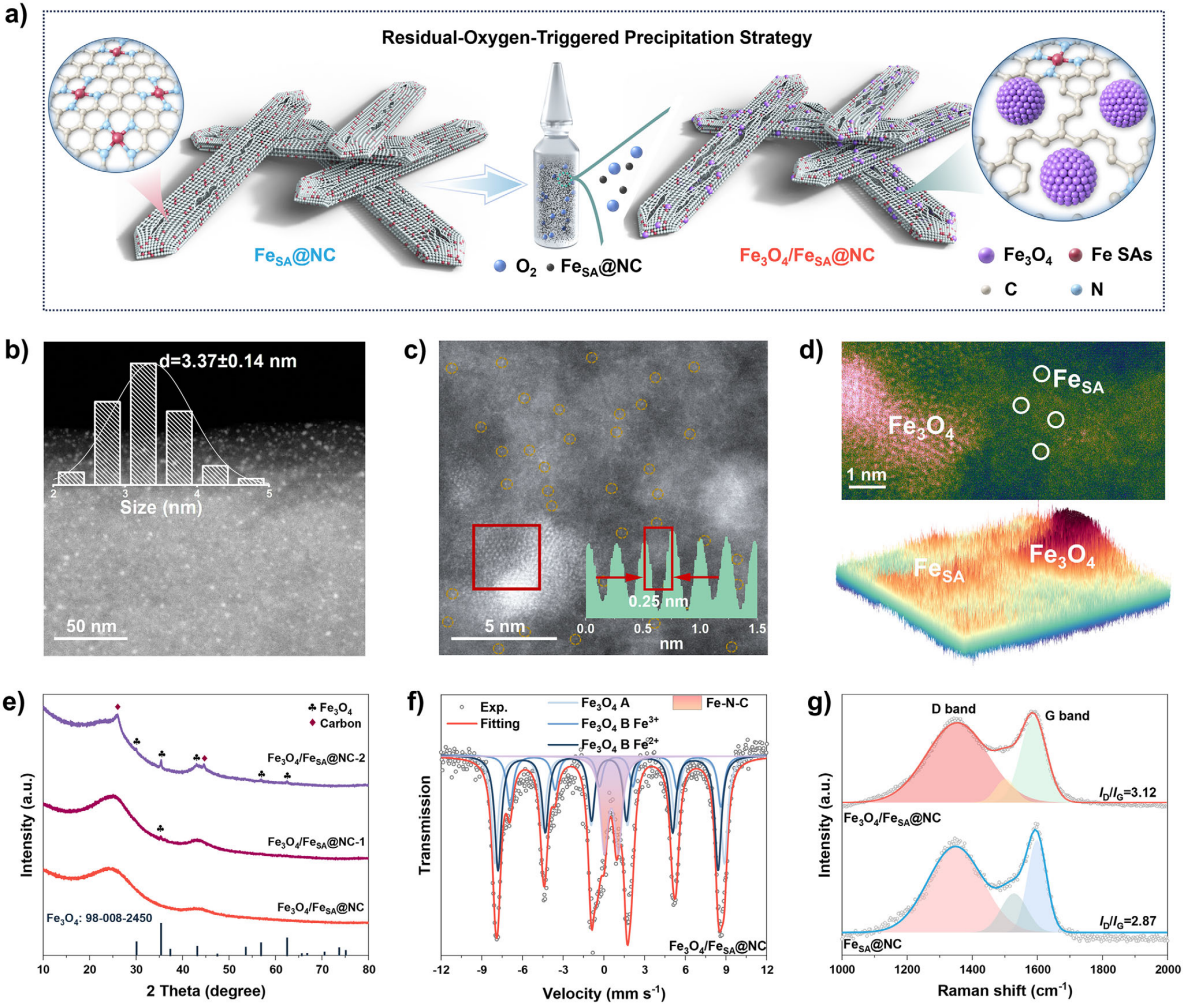
Synthesis and characterization of Fe_3_O_4_/Fe_SA_@NC nanorods. (a) Schematic of the synthesis process of Fe_SA_@NC and Fe_3_O_4_/Fe_SA_@NC. (b) HAADF‐STEM image and particle size statistics of Fe_3_O_4_/Fe_SA_@NC. (c) Aberration‐corrected HAADF‐STEM image of Fe_3_O_4_/Fe_SA_@NC and lattice fringes of Fe_3_O_4_‐cluster precipitates. (d) Pseudocolor HAADF‐STEM image of Fe_3_O_4_/Fe_SA_@NC showing the single‐atom/cluster distribution. (e) XRD patterns. (f) ^57^Fe Mössbauer spectra measured at 77 K of Fe_3_O_4_/Fe_SA_@NC. (g) Raman spectra.

The fabrication approach of Fe_3_O_4_/Fe_SA_@NC is illustrated in Figure 1a. Initially, uniform zeolitic imidazolate framework‐L (ZIF‐L) nanorods were fabricated via a facile liquid‐phase method. The ZIF‐L was then transformed into thermodynamically stable ZIF‐8 through a phase conversion process (Figures S1–S3), during which trace Fe‐containing species from Fe(NO_3_)_3_·9H_2_O were encapsulated into the ZIF‐8 framework, thereby providing the Fe source for the subsequent creation of single Fe‐atoms [28]. These nanorods were then converted into single‐atom Fe–N–C (Fe_SA_@NC) nanorods via a carbonization process that reduced and volatilized Zn species, accompanied by the creation of a defect‐rich, N‐doped carbon framework [29, 30]. The atomic dispersion of Fe in Fe_SA_@NC was confirmed by X‐ray diffraction (XRD) pattern (Figure S4a), which exhibited only two broad peaks of graphitic carbon at ∼25° and ∼44°. Meanwhile, aberration‐corrected high‐angle annular dark‐field scanning transmission electron microscope (AC‐HAADF‐STEM) images demonstrated that the Fe_SA_@NC nanorods contained exclusively isolated Fe atoms without any clusters (Figure S5). Finally, the synthesis of targeted Fe_3_O_4_/Fe_SA_@NC nanorods was achieved by purging Fe_SA_@NC with a N_2_/O_2_ mixture (20% O_2_), sealing under controlled vacuum pressures, and annealing at 800°C for 2 h (Figure S6). This controlled environment enabled the partial oxidation of Fe single‐atoms by trace oxygen to generate Fe_3_O_4_‐cluster precipitates, similar to the classic nanoprecipitation behavior in solid‐solution alloys [25].

Scanning electron microscope (SEM) images (Figure S4b) revealed that the Fe_3_O_4_/Fe_SA_@NC nanorods exhibited a highly porous structure, which could facilitate mass transport during catalytic reactions. Transmission electron microscope (TEM) images (Figure S4c) confirmed the well‐preserved rod‐like morphology with numerous cavities. A uniform distribution of Fe_3_O_4_‐cluster precipitates (average size: ∼3.4 nm) on the carbon matrix is demonstrated by the high‐magnification HAADF‐STEM image (Figures 1b and S7a). Elemental mapping (Figure S4d) demonstrated homogeneous distributions of C, N, and Fe, indicating uniform Fe incorporation throughout the N‐doped carbon matrix. AC‐HAADF‐STEM images (Figures 1c and S8) of Fe_3_O_4_/Fe_SA_@NC exhibited coexisting clusters and single atoms, bright regions (∼3 nm, marked by red rectangles) corresponding to clusters were clearly observed, with lattice fringes of ∼0.25 nm assigned to the (311) plane of the Fe_3_O_4_ phase (Figure S7b). To further definitively identify the iron oxide phase, FFT patterns (Figure S9) were performed from multiple HRTEM regions, showing the (022), (220), (202), and (400) planes of Fe_3_O_4_, and no spots matching Fe_3_C or α‐Fe_2_O_3_ were observed [31, 32]. Enlarged AC‐HAADF‐STEM image (Figure 1d) further revealed isolated Fe single atoms (white circles) dispersed around the nanoprecipitates, forming a cluster‐SAC hybrid catalyst. The EDS spectrum confirmed the Fe oxide‐based nature of nanoprecipitates (Figure S7c). Inductively coupled plasma optical emission spectrometry (Table S1) confirmed that the Fe content in Fe_3_O_4_/Fe_SA_@NC (∼0.81 wt%) is comparable to that in Fe_SA_@NC (∼0.82 wt%), indicating negligible metal loss during the secondary pyrolysis.

Similar to the XRD pattern of Fe_SA_@NC, no additional diffraction peaks from Fe‐containing species in Fe_3_O_4_/Fe_SA_@NC were discernible, indicating that the Fe species are atomically dispersed or present as ultrasmall clusters within the carbon matrix. The phase evolution of iron‐based nanoprecipitates was systematically investigated by controlling the residual oxygen content, achieved by engineering the vacuum level (10^−5^, 5 × 10^−5^, and 10^−4^ mbar) during secondary pyrolysis within sealed ampoules (Table S2). As shown in Figure 1e, a weak peak of Fe_3_O_4_/Fe_SA_@NC‐1 (synthesized at 5 × 10^−5^ mbar) emerges at ∼35.3°, which can be indexed to the (311) plane of the Fe_3_O_4_ phase (JCPDS 98‐008‐2450). Highly crystalline Fe_3_O_4_ grains can be observed in the TEM images of Fe_3_O_4_/Fe_SA_@NC‐1 (Figure S10). A distinct Fe_3_O_4_ phase is observed in Fe_3_O_4_/Fe_SA_@NC‐2, synthesized at a low vacuum of 10^−4^ mbar, which is attributed to excessive residual oxygen that severely etched the carbon matrix during secondary calcination. Consequently, TEM images reveal abundant ∼100 nm Fe_3_O_4_ crystals, and elemental mapping confirms the co‐localization of Fe and O (Figure S11), providing definitive validation of the Fe_3_O_4_ phase. In short, the vacuum level in the sealed ampoules is a critical determinant for the formation of Fe_3_O_4_‐cluster precipitates from Fe_SA_@NC. A sextet with isomer shift δ = 0.31 mm s^−1^ and hyperfine field H = 50.4 T in ^57^Fe Mössbauer spectra was a typical characteristic of the tetrahedral sites of Fe_3_O_4_ (Figure 1f and Table S3), further confirming that the nanoprecipitate phase in Fe_3_O_4_/Fe_SA_@NC is Fe_3_O_4_, not Fe_3_C or other Fe‐containing phases [33, 34]. Subsequently, the magnetic properties of Fe_3_O_4_/Fe_SA_@NC and Fe_SA_@NC were investigated using a vibrating sample magnetometer at 300 K (Figure S12). Notably, compared to Fe_SA_@NC, the Fe_3_O_4_/Fe_SA_@NC composite exhibits a typical hysteresis loop with enhanced saturation magnetization (*M*_s_, Fe_3_O_4_/Fe_SA_@NC: 0.24 emu/g, Fe_SA_@NC: 0.028 emu/g) and coercivity (*H*_c_, Fe_3_O_4_/Fe_SA_@NC: 405 Oe, Fe_SA_@NC: 21 Oe), resulting in a significantly enlarged hysteresis loop area. This finding provides clear evidence for the presence of magnetic Fe_3_O_4_ clusters in Fe_3_O_4_/Fe_SA_@NC.

**FIGURE 2:**
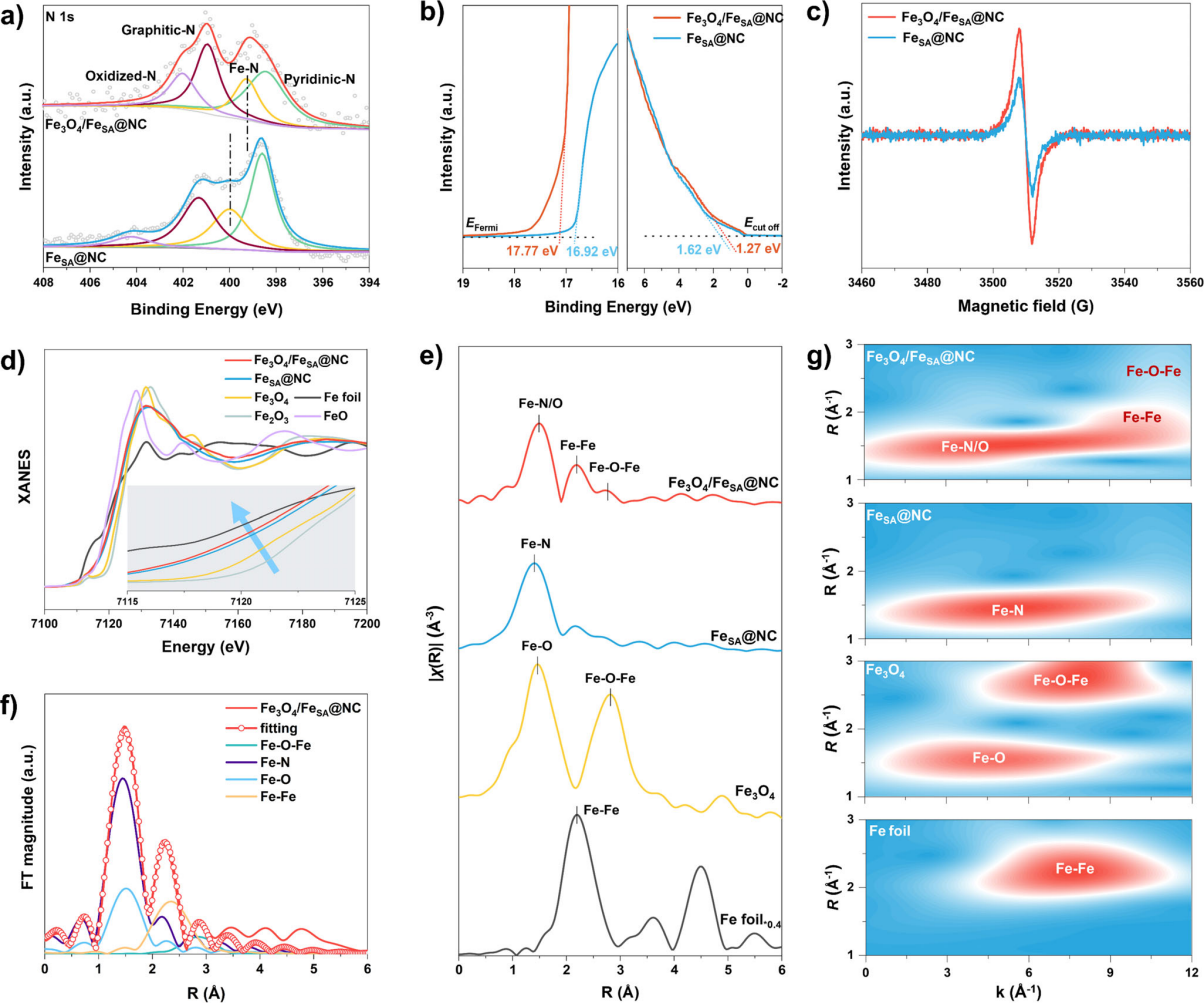
Fine structure characterization of the active sites. (a) XPS spectra of N 1s. (b) UPS spectra. (c) EPR spectra. (d) Normalized Fe K‐edge XANES spectra (inset is the magnified pre‐edge region). (e) Fourier transform k^3^‐weighted Fe K‐edge EXAFS spectra. (f) The corresponding EXAFS *R*‐space fitting curves of Fe_3_O_4_/Fe_SA_@NC. (g) Wavelet transform of Fe_3_O_4_/Fe_SA_@NC, Fe_SA_@NC, Fe_3_O_4_ and Fe foil.

Raman spectra (Figure 1g) exhibited characteristic peaks at 1350 cm^−1^ (D‐band, associated with lattice defects) and 1600 cm^−1^ (G‐band, corresponding to *sp* ^2^‐hybridized carbon). The area ratio (*I*_D_/*I*_G_) of Fe_3_O_4_/Fe_SA_@NC (3.12) was significantly higher than that of Fe_SA_@NC (2.87), indicating a greater abundance of defective carbon nanostructures in the Fe_3_O_4_/Fe_SA_@NC matrix. Brunauer–Emmett–Teller (BET) surface area analysis (Figure S13) revealed that Fe_3_O_4_/Fe_SA_@NC possessed a substantially larger specific surface area (1120.8 m^2^ g^−1^) compared with Fe_SA_@NC (810.3 m^2^ g^−1^). The increased mesopores observed in Fe_3_O_4_/Fe_SA_@NC, resulting from residual oxygen etching in sealed ampoules, lead to enhanced reactant penetration and product transport during the catalytic process. X‐ray photoelectron spectroscopy (XPS) was employed to investigate the electronic and coordination structures of the catalysts (Figures 2a and S14–S17). The presence of C─N bonds in both Fe_3_O_4_/Fe_SA_@NC and Fe_SA_@NC confirms that the carbon matrix is nitrogen‐doped. Compared to the Fe_SA_@NC, the observed lower binding energy shift (∼0.9 eV) of the Fe─N bond in Fe_3_O_4_/Fe_SA_@NC demonstrates that the Fe_3_O_4_‐cluster precipitates induce a strong electronic perturbation, leading to an asymmetric electron density distribution in the Fe‐N_4_ moieties (Figure 2a). Remarkably, Fe_3_O_4_/Fe_SA_@NC demonstrated a higher content of pyridinic‐N species (Table S4), which might reduce the energy barrier for O_2_ adsorption on adjacent carbon atoms and serve as effective active sites to accelerate the formation of oxygen‐containing intermediates during ORR [35, 36].

The observed binding energy reduction in Fe^2+^ 2p_1/2_ indicates disruption of Fe‐N_4_ symmetry and electron transfer to the carbon substrate, while no detectable Fe^3+^ signals were observed due to the low Fe content in Fe_3_O_4_/Fe_SA_@NC (Figure S16). Furthermore, ultraviolet photoelectron spectroscopy (UPS) analysis showed that Fe_3_O_4_/Fe_SA_@NC possessed a higher work function (14.6 eV) than Fe_SA_@NC (14.2 eV) (Figures 2b and S18), suggesting an enhanced electron donation capability to ORR intermediates. These comprehensive spectroscopic analyses collectively reveal that the Fe_3_O_4_‐cluster precipitates modulate the electronic structure of Fe‐N_4_ centers while the abundant pyridinic‐N species provide additional active sites, with the modified work function further promoting electron transfer. Complementary electron paramagnetic resonance (EPR) spectroscopy revealed significant changes in the number of unpaired electrons and chemical environments of Fe single‐atoms (Figure 2c). The peak‐to‐peak linewidth increases from approximately 2.8 to 4.1 G upon incorporation of Fe_3_O_4_ precipitates, suggesting enhanced spin‐spin relaxation due to magnetic interaction between the Fe‐N_4_ sites and Fe_3_O_4_ clusters. The integrated EPR intensity is approximately 1.9 times higher for Fe_3_O_4_/Fe_SA_@NC, indicating a higher concentration of unpaired electrons, consistent with electron donation from Fe_3_O_4_ to Fe–N_4_ centers.

X‐ray absorption fine structure (XAFS) analysis provided deep insight into the local electronic structure of Fe atoms in Fe_3_O_4_/Fe_SA_@NC. The X‐ray absorption near‐edge structure (XANES) at Fe K‐edge showed that the absorption energy of Fe_3_O_4_/Fe_SA_@NC is situated between those of FeO and Fe_3_O_4_ (Figure 2d). It was noteworthy that the Fe K edge of Fe_3_O_4_/Fe_SA_@NC shifts to lower energy compared to Fe_SA_@NC, suggesting a lower average Fe oxidation state. This trend is counterintuitive given the higher formal oxidation state of Fe in Fe_3_O_4_. We attribute this phenomenon to interfacial electron transfer from Fe_3_O_4_ clusters to the adjacent Fe‐N_4_ sites. The Fermi level equilibration across the heterointerface results in electron accumulation on the Fe‐N_4_ centers, which dominate the total Fe content. This result is consistent with XPS observations of asymmetric electron density distribution in Fe‐N_4_ sites induced by Fe_3_O_4_‐cluster precipitates. Fourier‐transform extended XAFS (FT‐EXAFS) analysis revealed the crucial coordination information of Fe species (Figure 2e). Both Fe_3_O_4_/Fe_SA_@NC and Fe_SA_@NC displayed a prominent peak at ∼1.4 Å corresponding to Fe–N coordination, while Fe_3_O_4_/Fe_SA_@NC exhibited additional Fe–Fe and Fe–O–Fe scattering paths. Quantitative EXAFS fitting analysis (Figures 2f, S19, and S20; Tables S5 and S6) unlocked the coexistence of atomically dispersed Fe atoms in Fe‐N_4_ configuration alongside Fe_3_O_4_ clusters featuring Fe–O–Fe and Fe–O coordination. The additional Fe–Fe signals indicated that partial Fe–N_4_ sites were likely bridged to Fe_3_O_4_ clusters through Fe─Fe bonds. Moreover, the coordination number of Fe─N bonds in Fe_SA_@NC is ∼3.83, indicating the formation of atomic Fe–N_4_ sites (Figure S21 and Table S7). Wavelet transform (WT) EXAFS analysis (Figure 2g) provided further coordination information on Fe species. The intensity maximum at ∼5.5 Å^−1^ in both samples confirmed the Fe–N coordination, while additional features at ∼6.9 Å^−1^ and ∼10.1 Å^−1^ in Fe_3_O_4_/Fe_SA_@NC unequivocally verified Fe–Fe and Fe–O–Fe coordination. The subtle positional differences in the Fe–N WT maxima further evidenced the strong electronic coupling between the Fe–N_4_ sites and Fe_3_O_4_‐cluster precipitates. Collectively, these structural characterizations demonstrate that Fe_3_O_4_/Fe_SA_@NC represents a composite architecture combining atomic Fe‐N_4_ sites with Fe_3_O_4_‐cluster precipitates, in contrast to Fe_SA_@NC containing only isolated Fe‐N_4_ sites.

### 2.2 Electrocatalytic Performance of Fe_3_O_4_/Fe_SA_@Nc Catalysts

**FIGURE 3:**
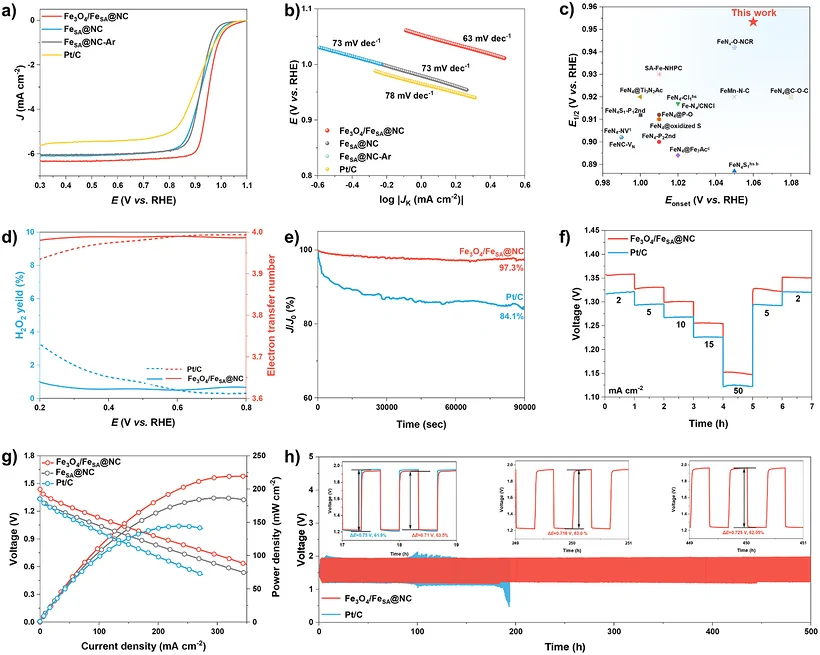
Electrocatalytic performance evaluation of Fe_3_O_4_/Fe_SA_@NC. (a) LSV curves of Fe_3_O_4_/Fe_SA_@NC, Fe_SA_@NC, Fe_SA_@NC‐Ar, and 20% Pt/C in 0.1 M KOH. (b) Tafel slopes for the corresponding catalysts. (c) Comparison of ORR performance with advanced Fe–N–C‐based electrocatalysts in alkaline conditions. (d) H_2_O_2_ yield and electron transfer number of Fe_3_O_4_/Fe_SA_@NC and Pt/C. (e) Chronoamperometric stability test of Fe_3_O_4_/Fe_SA_@NC and Pt/C. (f) Rate curves at different current densities of Fe_3_O_4_/Fe_SA_@NC and commercial Pt/C based Zn‐air battery. (g) Discharge polarization curves and the corresponding power density of Fe_3_O_4_/Fe_SA_@NC, Fe_SA_@NC, and Pt/C in ZABs. (h) Charge‐discharge cycling curves of rechargeable ZABs using Fe_3_O_4_/Fe_SA_@NC and Pt/C as cathode catalysts.

The ORR performance of Fe_3_O_4_/Fe_SA_@NC nanorods was evaluated using rotating disk electrode (RDE) and rotating ring disk electrode (RRDE) techniques in O_2_‐saturated 0.1 M KOH electrolyte, with commercial 20% Pt/C, Fe_SA_@NC, and Fe_SA_@NC‐Ar as reference samples. Linear sweep voltammetry (LSV) curves (Figure 3a) revealed that Fe_3_O_4_/Fe_SA_@NC exhibited an onset potential (*E*_onset_) of 1.06 V versus RHE and an ultrahigh half‐wave potential (*E*_1/2_) of 0.953 V versus RHE, while Fe_SA_@NC showed a downward *E*_1/2_ of 0.920 V. Notably, the *E*_1/2_ of Fe_SA_@NC‐Ar remained at 0.920 V, confirming that secondary pyrolysis alone does not affect the ORR activity. Moreover, the *E*_1/2_ of bare NC nanorods containing trace Zn (<0.3 wt%) is only 0.82 V, revealing the decisive role of Fe‐containing species on ORR activity of Fe_3_O_4_/Fe_SA_@NC and Fe_SA_@NC (Figure S22). These results demonstrate that the presence of Fe_3_O_4_‐cluster precipitates can significantly enhance the ORR activity of Fe_SA_@NC. Fe_3_O_4_/Fe_SA_@NC achieved the lowest value of 63.2 mV dec^−1^, compared to Fe_SA_@NC (73.8 mV dec^−1^) and Pt/C (78.4 mV dec^−1^), indicating that the Fe_3_O_4_‐cluster precipitates significantly accelerate the ORR kinetics of Fe‐N_4_ sites (Figure 3b). Additionally, the kinetic current density (*J*_k_) reflects the intrinsic rate of the surface reaction on the catalyst. Fe_3_O_4_/Fe_SA_@NC delivered ultrahigh *J*_k_ of 135.3 mA cm^−2^ and 92.4 mA cm^−2^ at 0.85 and 0.9 V, respectively, significantly surpassing those of Fe_SA_@NC and commercial Pt/C (Figure S23). In brief, the ORR activity of coupled Fe_3_O_4_/Fe_SA_@NC places it among the top‐performing, advanced Fe–N–C‐based catalysts (Figure 3c and Table S8) [9, 11, 14, 16, 18, 37, 38, 39, 40, 41, 42, 43, 44].

Notably, the secondary pyrolysis temperature of the carbon support critically influences its graphitization degree, defect density, and thus its ORR activity. The optimal ORR activity was achieved with Fe_3_O_4_/Fe_SA_@NC pyrolyzed at 800°C, which surpassed those prepared at 600°C, 700°C, and 900°C by delivering a more positive *E*_1/2_ (Figure S24). The Fe(NO_3_)_3_·9H_2_O dosage relevant to the Fe content of targeted Fe_3_O_4_/Fe_SA_@NC is also important for the ORR activities (Figure S25). The secondary pyrolysis pressure directly affected the residual oxygen content in the carbon matrix, thereby governing the distribution and size of Fe_3_O_4_ phases. Pyrolysis at 10^−5^ mbar produced the most active Fe_3_O_4_/Fe_SA_@NC catalyst (Figure S26). The Koutecky–Levich (K–L) analysis of LSV curves recorded at 900–2500 rpm revealed an average electron transfer number (*n*) of ∼4.0 for Fe_3_O_4_/Fe_SA_@NC, confirming a dominant 4e^−^ pathway for O_2_ reduction to H_2_O (Figure S27). RRDE tests further validated this mechanism, with H_2_O_2_ yields below 2% and *n* values between 3.98 and 4.0 in the potential window of 0.4–0.8 V (Figure 3d). Notably, in sharp contrast to the Pt/C catalyst, the Fe_3_O_4_/Fe_SA_@NC demonstrated exceptional stability with only a 2.7% current density loss after 25 h durability testing (Figure 3e) and methanol tolerance (Figure S28), underscoring its potential for practical applications.

Given the remarkable ORR performance, Fe_3_O_4_/Fe_SA_@NC nanorods were employed as air‐cathode catalysts in ZABs. The assembled battery achieved a high open‐circuit voltage (OCV) of 1.53 V, which is higher than that of Pt/C (∼1.46 V, Figure S29). Notably, the Fe_3_O_4_/Fe_SA_@NC based ZAB exhibited consistently higher discharge voltages than Pt/C across a range of current densities (2–50 mA cm^−2^), highlighting its excellent ORR activity (Figure 3f). The specific capacity reached 817.6 mAh g^−1^ at 20 mA cm^−2^, surpassing that of Pt/C (790.4 mAh g^−1^, Figure S30). Moreover, the maximum power density of the Fe_3_O_4_/Fe_SA_@NC based ZAB was 219.4 mW cm^−2^, significantly outperforming the Fe_SA_@NC (185.4 mW cm^−2^) and commercial Pt/C (148.2 mW cm^−2^, Figure 3g). The Fe_3_O_4_/Fe_SA_@NC cathode demonstrated an impressive round‐trip efficiency of 63.5% (Figure 3h). Even after 500 h of cycling, the charge‐discharge voltage gap increased by only 15 mV, highlighting exceptional long‐term stability. In contrast, Pt/C‐based ZABs exhibited rapid performance degradation after ∼100 h.

**FIGURE 4:**
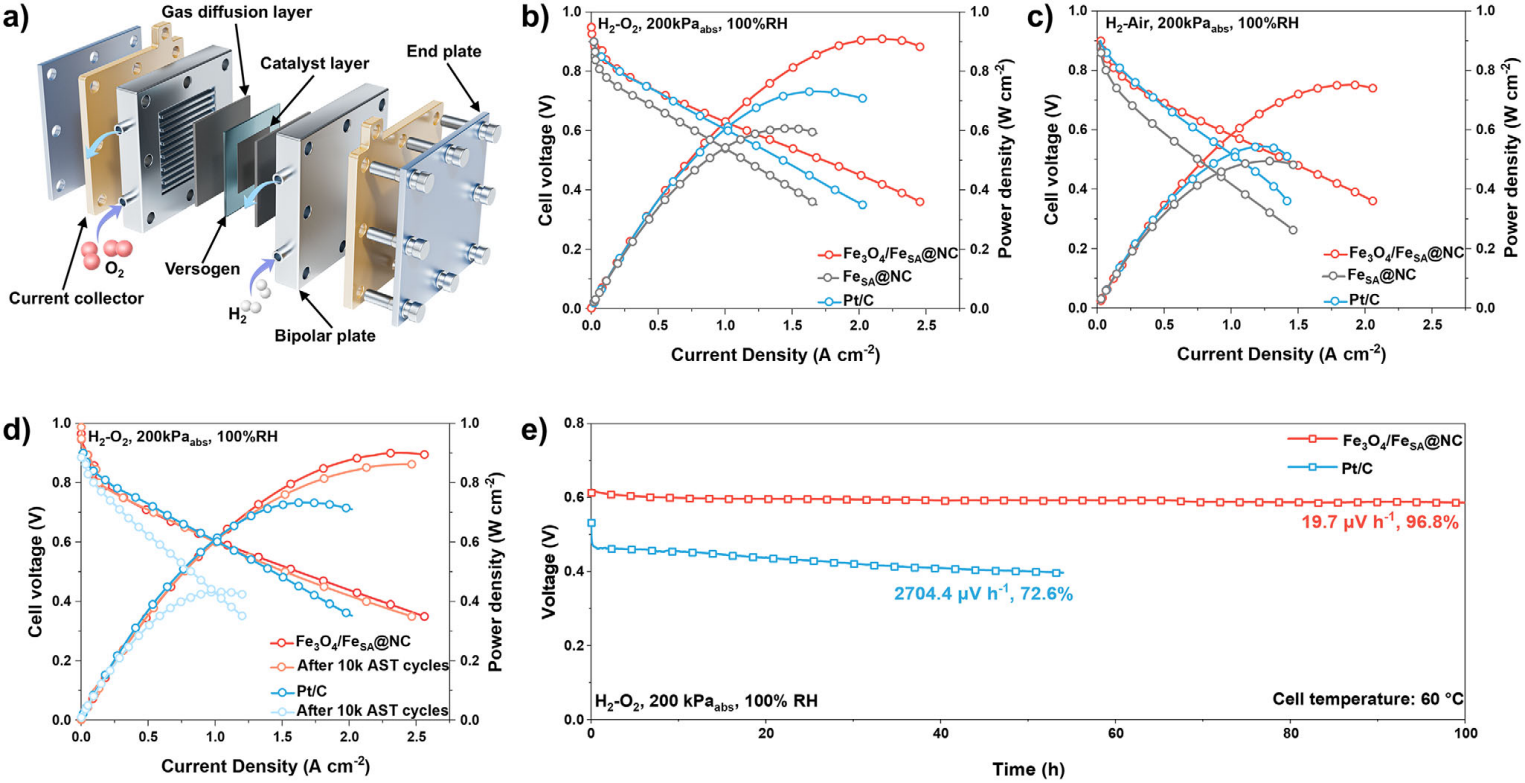
AEMFC performance. (a) Schematic illustration of the AEMFC. (b) AEMFC performance obtained by using Fe_3_O_4_/Fe_SA_@NC, Fe_SA_@NC and commercial Pt/C as cathode catalyst. (c) AEMFC performance obtained by using Fe_3_O_4_/Fe_SA_@NC, Fe_SA_@NC, and commercial Pt/C as cathode when the cathode gas was CO_2_‐free dry air (20% O_2_). (d) AST performance of Fe_3_O_4_/Fe_SA_@NC and Pt/C. (e) AEMFC stability performance obtained by using Fe_3_O_4_/Fe_SA_@NC and Pt/C as cathode catalyst.

To further evaluate practical applicability, AEMFCs were assembled using Fe_3_O_4_/Fe_SA_@NC, Fe_SA_@NC, and Pt/C as cathode catalysts (Figure 4a). The Fe_3_O_4_/Fe_SA_@NC based AEMFC (Figure 4b) achieved a high peak power density (PPD) of 909.3 mW cm^−2^ at 2.17 A cm^−2^, far exceeding those of Fe_SA_@NC (606.8 mW cm^−2^ at 1.45 A cm^−2^) and Pt/C (733.2 mW cm^−2^ at 1.67 A cm^−2^). When the cathode gas was switched to CO_2_‐free air, an impressive PPD of 751.4 mW cm^−2^ at 1.87 A cm^−2^ can be obtained for Fe_3_O_4_/Fe_SA_@NC based AEMFC, significantly surpassing the performance of Fe_SA_@NC (493.1 mW cm^−2^ at 1.25 A cm^−2^) and Pt/C (543.9 mW cm^−2^ at 1.22 A cm^−2^). This remarkable enhancement demonstrates the superior catalytic efficiency of Fe_3_O_4_/Fe_SA_@NC even under realistic, oxygen‐limited operating conditions (Figure 4c). Moreover, the Fe_3_O_4_‐cluster precipitates can enhance the stability of Fe‐N‐C SACs. The accelerated stress test (AST) is a universal standard protocol for evaluating catalyst stability, with the detailed procedure illustrated in Figure S31. After 10 000 AST cycles, Fe_3_O_4_/Fe_SA_@NC exhibits only a marginal activity decay of approximately 4.2% under H_2_–O_2_ conditions (Figure 4c), in sharp contrast to the significant 41.6% decay observed for Pt/C (Figure 4c) and 20.3% decay for Fe_SA_@NC (Figure S32), demonstrating the outstanding long‐term durability of Fe_3_O_4_/Fe_SA_@NC based AEMFCs. Notably, the Fe_3_O_4_/Fe_SA_@NC based AEMFC demonstrated exceptional durability, maintaining 96.8% voltage retention after 100 h of continuous operation at 0.5 A cm^−2^, with a voltage decay rate of only 19.7 µV h^−1^ (Figure S33a). This performance far surpassed that of the Pt/C based AEMFC (2704.4 µV h^−1^), further confirming the superior long‐term stability of the Fe_3_O_4_/Fe_SA_@NC cathode catalyst (Figures 4e and S33b). The AEMFC performance of Fe_3_O_4_/Fe_SA_@NC was also compared with state‐of‐the‐art cathode electrocatalysts (Figure S34), clearly highlighting its superior peak power density and confirming the effectiveness of our modulation strategy (Table S9) [4, 45, 46, 47, 48, 49, 50, 51, 52, 53, 54, 55, 56].

### 2.3 The Role of Fe_3_O_4_‐Cluster Precipitates on Enhanced ORR Activity in Fe_3_O_4_/Fe_SA_@NC

**FIGURE 5:**
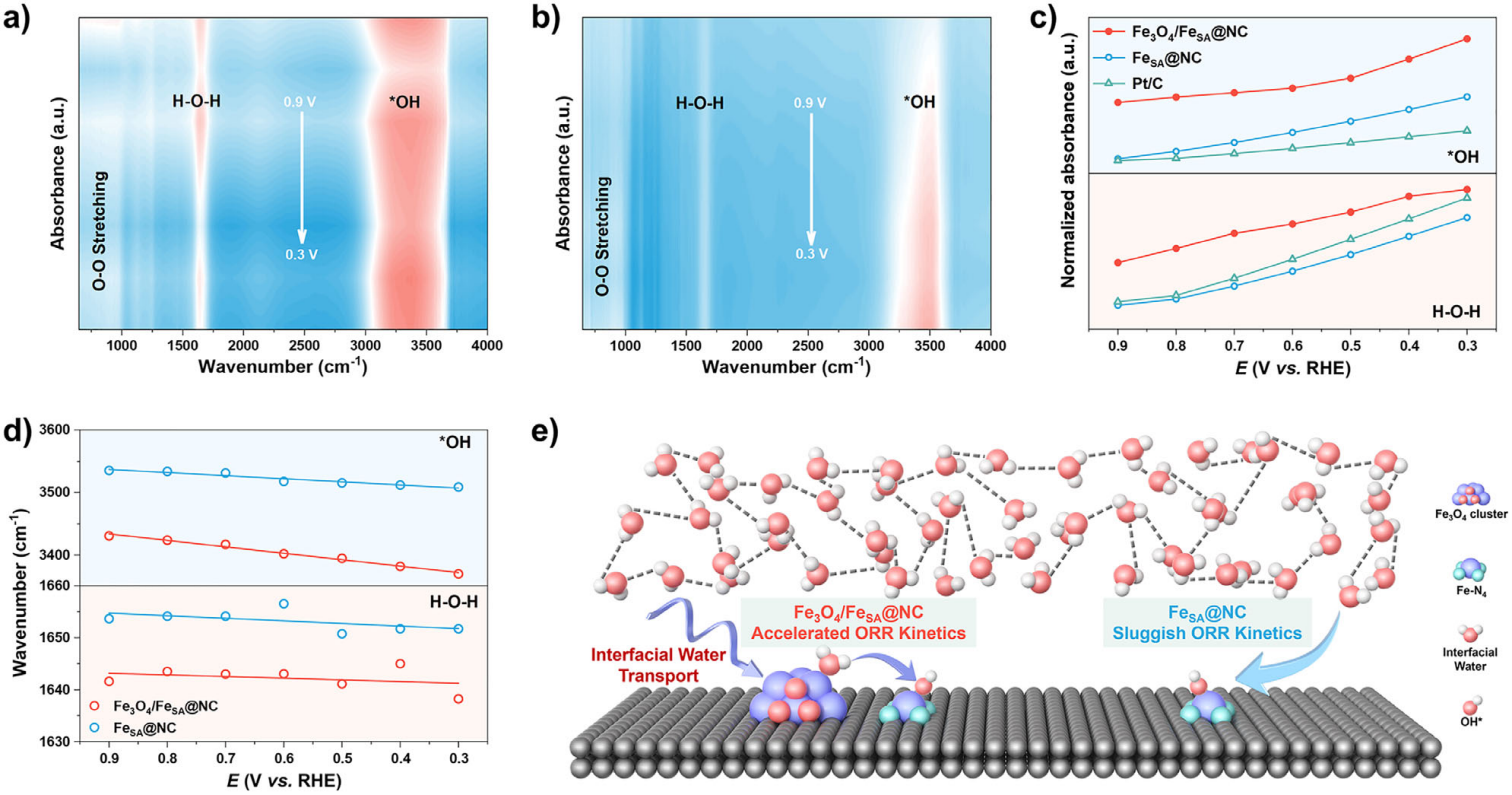
In situ characterization and reaction mechanism investigation of the alkaline ORR on Fe_3_O_4_/Fe_SA_@NC and Fe_SA_@NC. In situ ATR‐SEIRAS of (a) Fe_3_O_4_/Fe_SA_@NC and (b) Fe_SA_@NC catalysts during the ORR. (c) The detailed variations of H–O–H and *O‐H with potential. (d) The variations of wavenumber with potential. (e) Schematic illustration of the water dissociation mechanism for Fe_3_O_4_/Fe_SA_@NC and Fe_SA_@NC.

In situ ATR‐SEIRAS was employed to probe the interfacial water structure and elucidate the accelerated water dissociation mechanism. As shown in Figures 5a,b and S35, characteristic vibrational modes of interfacial water were observed, including the H–O–H bending mode (1600–1700 cm^−1^) and *OH stretching frequency (3300–3600 cm^−1^), confirming the direct participation of water in the ORR process [16]. Crucially, Fe_3_O_4_/Fe_SA_@NC exhibited a clear *OOH signal at ∼1250 cm^−1^ with no detectable HOOH* peak within the range from 0.9 to 0.3 V (Figure S36), suggesting the preferred 4e^−^ ORR pathway [57]. Notably, the intensities of both H–O–H bending and *OH stretching modes were significantly enhanced on Fe_3_O_4_/Fe_SA_@NC, compared to Fe_SA_@NC at all applied potentials (Figure 5c), indicating that Fe_3_O_4_‐cluster precipitates significantly enhance the accumulation and subsequent dissociation of interface water, consistent with a promoted water‐dissociation process [58]. Furthermore, Fe_3_O_4_/Fe_SA_@NC exhibited substantially stronger *OH peak intensities at low potentials, likely due to Fe_3_O_4_ clusters facilitating partial *OH grafting on Fe‐N_4_ sites. The accumulation of *OH species at low potentials, likely stabilized through interaction with Fe_3_O_4_‐derived sites, may facilitate subsequent *OH desorption steps and thereby contribute to the enhanced ORR kinetics. To probe the proton transfer kinetics, we compared the ORR activities of Fe_3_O_4_/Fe_SA_@NC and Fe_SA_@NC in H_2_O‐ and D_2_O‐ based 0.1 M KOH electrolytes. As shown in Figure S37, the smaller kinetic isotope effect (KIE, ∼1.41) of Fe_3_O_4_/FeSA@NC than Fe_SA_@NC (∼1.92) suggested that the introduction of Fe_3_O_4_ clusters can substantially alleviate the PCET limitation [59]. Comparative analysis with commercial Pt/C revealed much higher stretching intensities of H–O–H and *OH for both Fe‐based catalysts, underscoring the superiority of Fe–N–C SACs for alkaline ORRs. Importantly, the lower wavenumber of interfacial H–O–H bending vibration on Fe_3_O_4_/Fe_SA_@NC further confirms the enhanced hydrogen bond strength in the presence of Fe_3_O_4_‐cluster precipitates (Figures 5d and S38a). The *OH stretching peak of Fe_3_O_4_/Fe_SA_@NC (∼3400 cm^−1^) exhibited a 100 cm^−1^ redshift relative to Fe_SA_@NC (∼3500 cm^−1^), indicating stronger hydrogen bonding between interfacial water molecules and oxygen intermediates (Figure S38b). Since the proton transfer occurs primarily via the Grotthuss hopping mechanism through extended hydrogen‐bond networks, the strong hydrogen‐bond networks would enhance the proton transfer kinetics [60]. Taken together, the reduced KIE and the red‐shifted *OH stretching frequency are consistent with the formation of a strongly hydrogen‐bonded interfacial water network in Fe_3_O_4_/Fe_SA_@NC. The Fe_3_O_4_‐cluster precipitates would promote water dissociation to generate protons and rapidly transport them to the active Fe‐N_4_ sites through the enhanced hydrogen‐bond networks, thereby contributing to the accelerated PCET kinetics (Figures 5e and S39) [61, 62].

**FIGURE 6:**
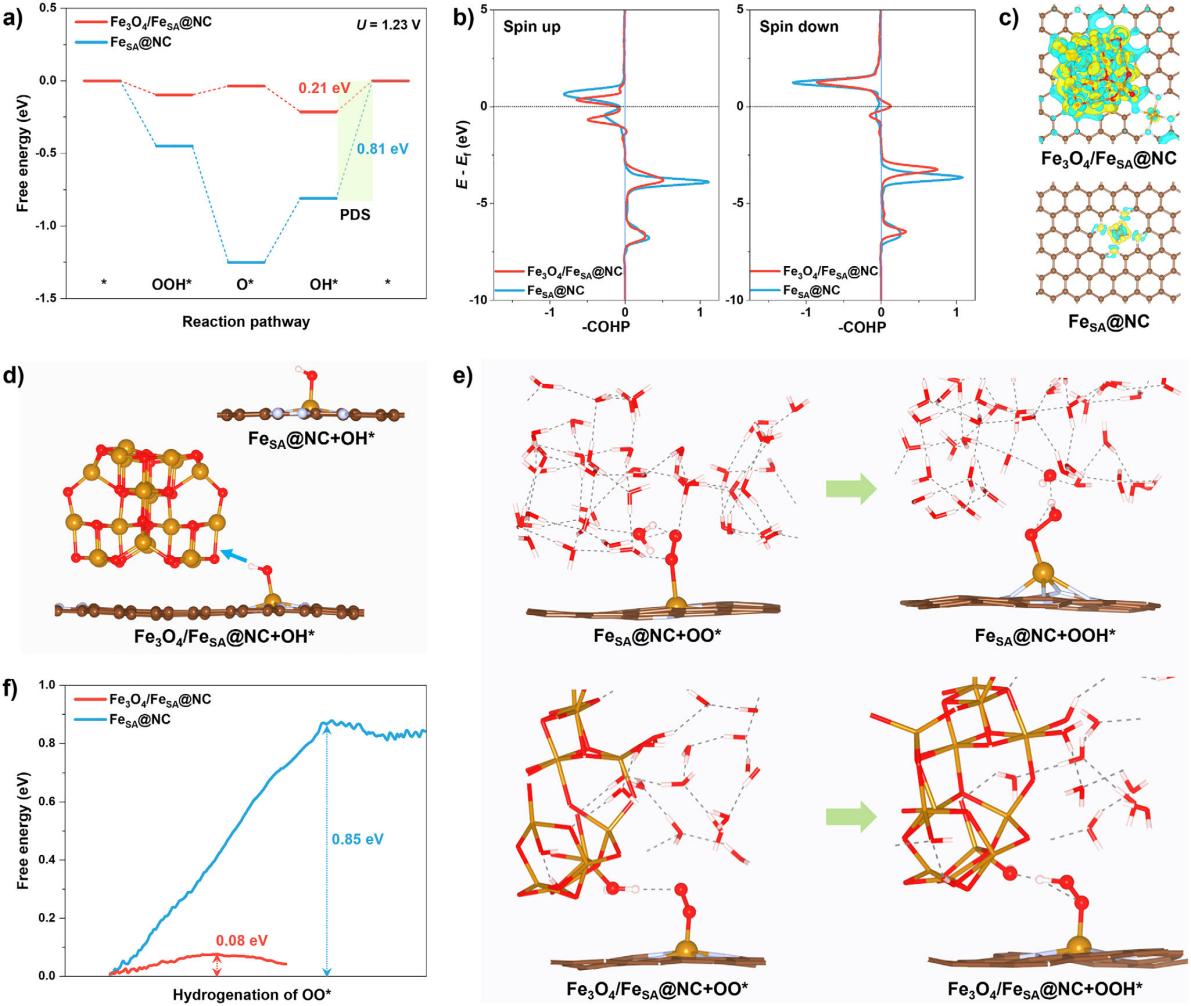
Theoretical calculation of the catalytic activity. (a) Computed ORR free energy profile for Fe_3_O_4_/Fe_SA_@NC and Fe_SA_@NC at U = 1.23 V (where the PDS is labeled), respectively. (b) Computed COHP of the hybridizations between Fe 3d_z_^2^ and O 2p_z_ orbitals for OH* adsorbed on Fe_3_O_4_/Fe_SA_@NC and Fe_SA_@NC, respectively (The dashed lines indicate the Fermi level). (c) The differential charge density of Fe_3_O_4_/Fe_SA_@NC and Fe_SA_@NC (yellow and blue clouds indicate the accumulation and depletion of electron density, respectively). (d) The optimized structures of OH* adsorbed on Fe_3_O_4_/Fe_SA_@NC and Fe_SA_@NC (The orange, brown, blue, red, and white balls indicate the Fe, C, N, O, and H, respectively). (e) Representative snapshots of hydrogenation of OO* on Fe_SA_@NC (upper panel) and Fe_3_O_4_/Fe_SA_@NC (bottom panel). (f) The calculated free‐energy barrier of hydrogenation of OO* on Fe_SA_@NC and Fe_3_O_4_/Fe_SA_@NC.

Density functional theory (DFT) calculations were performed to identify the electronic origin of the enhanced ORR activity of Fe_3_O_4_/Fe_SA_@NC. Based on the structural measurement, two simulation models, namely, Fe_SA_@NC and Fe_3_O_4_/Fe_SA_@NC, were built (Figures S40 and S41). The energy profiles of the ORR with a four‐electron pathway involving intermediates OOH*, O*, and OH* on the active site (Fe single atom bonded to N‐C) were calculated (Figure 6a). Under *U* = 1.23 V, the fourth step (OH* + e^−^ → * + OH^−^) exhibits the largest change in free energy for both Fe_SA_@NC and Fe_3_O_4_/Fe_SA_@NC, indicating that OH* desorption from the active site is the potential‐determining step (PDS). Notably, with a supported Fe_3_O_4_ cluster nearby the active site, the affinity of the Fe active site towards all intermediates (OOH*, O*, and OH*) is weakened, which is consistent with the spectroscopic signature observed in ATR‐SEIRAS. The free energy change of the PDS for Fe_3_O_4_/Fe_SA_@NC is only 0.21 eV, significantly lower than that for Fe_SA_@NC (0.81 eV). To reveal the effects of the Fe_3_O_4_ cluster on the intrinsic catalytic activity for ORR on the Fe single site, the electronic properties, including population analysis, projected density of states (PDOS), and crystal orbital Hamilton population (COHP) analysis, were calculated (Figure 6b). In general, the interaction between the active center Fe and intermediate OH* is dominated by the hybridization between Fe 3d_z_^2^ and O 2p_z_ orbitals, forming an Fe─O bond perpendicular to the carbon support (Figures 6d and S42). In contrast, the incorporation of Fe_3_O_4_ cluster nearby breaks the symmetric electron density distribution in the Fe_SA_@NC moiety (Figures 6c and S42c; Table S10), which is consistent with XPS results. This asymmetric electron density distribution leads to adsorbed OH* tilted towards the neighboring Fe_3_O_4_ cluster, thereby reducing the orbital overlapping between Fe 3d_z_^2^ and O 2p_z_ in the vertical direction and weakening the adsorption of OH* (ICOHP values: Fe_SA_@NC −0.61 eV vs. Fe_3_O_4_/Fe_SA_@NC −0.41 eV). Furthermore, the introduction of the Fe_3_O_4_ cluster slightly lowers the oxidation state and downshifts the d‐band center (*ε*_d_) of the Fe single site (Figure S42d). The former agrees well with XANES results, while the latter optimizes the binding strength of OH*, leading to enhanced ORR performance.

Ab initio molecular dynamics (AIMD) simulations were further performed to explore the contribution of the Fe_3_O_4_ cluster to the water hydrolysis‐associated PCET process during ORR. As illustrated in Figure 6e (upper panel), a typical hydrogen‐bond network forms in the Fe–N–C/OO*/water interface, and the calculated free‐energy barrier of hydrogenation of OO* on Fe sites is 0.85 eV at 1.0 V versus RHE (Figure 6f). On the other hand, as shown in Figure 6e (bottom panel), partial Fe sites of the Fe_3_O_4_ cluster are covered by water molecules, while O sites form a hydrogen bond with interfacial water. The H─O bond in adsorbed H_2_O* continuously forms and breaks under interfacial fluctuations, which effectively lowers the kinetic barrier for OO* protonation to 0.08 eV (Figure 6f). Overall, the combined theoretical and experimental analysis shows that the incorporation of a nearby Fe_3_O_4_ cluster not only induces an asymmetric electronic structure of the Fe‐N_4_ active center to weaken intermediate adsorption but also affects the interfacial hydrogen‐bond network and proton transfer to accelerate the kinetics of the PCET process, both benefiting the ORR. Impressively, the enhanced water dissociation associated with PCET steps ensures a rapid and continuous proton supply, effectively mitigating the water‐deficient issue and enabling the outstanding PPD and durability of Fe_3_O_4_/FeSA@NC based AEMFC devices.

## 3 Conclusion

In summary, Fe_3_O_4_ clusters can be precipitated onto Fe‐N‐C SACs through a residual‐oxygen‐assisted precipitation approach. The in situ generated Fe_3_O_4_‐cluster precipitates can effectively modulate the electronic structure of the nearby active Fe‐N_4_ sites to boost the sluggish ORR kinetics. *Operando* spectroscopy and ab initio calculations collectively demonstrate that Fe_3_O_4_‐cluster precipitates not only induce an asymmetric electron distribution in Fe‐N_4_ active sites–reducing the energy barriers of all ORR intermediates, particularly *OH–but also accelerate water dissociation and the associated PCET processes, thereby significantly speeding up the ORR kinetics. Notably, the coupled Fe_3_O_4_/Fe_SA_@NC catalyst yields exceptional ORR performance in 0.1 M KOH electrolyte, including a highly positive *E*_1/2_ of 0.953 V and an ultrahigh *J*_K_ of 135.3 mA cm^−2^ at 0.85 V. When deployed as cathode catalysts in ZABs and AEMFCs, Fe_3_O_4_/Fe_SA_@NC yields high peak power density and long‐term durability under operational conditions. This work establishes a generic approach for in situ fabrication of metal oxide clusters to assist SACs by harnessing residual oxygen, offering a design strategy for high‐performance electrocatalysts across diverse chemical applications.

## Author Contributions

**Cheng‐Kai Du**: investigation, methodology, conceptualization, data curation, visualization. **Xiongyi Liang**: writing – original draft, investigation, software, data curation, validation, visualization. **Fei‐Xiang Ma**: supervision, conceptualization, methodology, investigation, funding acquisition, writing – review and editing, formal analysis, project administration, visualization. **Yutong Li**: investigation, methodology. **Zheng‐Qi Liu**: investigation, methodology. **Long Ma**: investigation. **Zeng Li**: investigation. **Liang Zhen**: resources, supervision. **Yan Huang**: supervision, writing – review and editing. **Xiao Cheng Zeng**: writing – review and editing, resources, supervision, software, project administration. **Cheng‐Yan Xu**: writing – review and editing, supervision, funding acquisition, conceptualization, project administration.

## Conflicts of Interest

The authors declare no conflicts of interest

## Supporting information




**Supporting File**: anie73518‐sup‐0001‐SuppMat.docx.

## Data Availability

The data that support the findings of this study are available in the supporting Information of this article.
