# Supplementary material for: Coupled Fe3O4‐Cluster Precipitates and Single Fe‐Atom Catalysts Can Boost Oxygen Reduction via Concomitantly Accelerated Water Dissociation
Source: Angew Chem Int Ed Engl. 2026 Jul 9;65(37):e5493485. doi: 10.1002/anie.5493485 (PMC13549053; doi:10.1002/anie.5493485)
Supplement: Supplementary file 1 [file ANIE-65-e5493485-s001.docx]

**Supporting Information**

**Coupled Fe_3_O_4_-Cluster Precipitates and Single Fe-Atom Catalysts Can Boost Oxygen Reduction via Concomitantly Accelerated Water Dissociation**

*Cheng-Kai Du^#^, Xiongyi Liang^#^, Fei-Xiang Ma*, Yutong Li, Zheng-Qi Liu, Long Ma, Zeng Li, Liang Zhen, Yan Huang, Xiao Cheng Zeng*, Cheng-Yan Xu**

Email: mafeixiang@hit.edu.cn; xzeng26@cityu.edu.hk; cy_xu@hit.edu.cn

**Experimental Section**

**Synthesis of Fe_SA_@NC nanorods**

Typically, 3.0 g of 2-methylimidazole was dissolved in 120 mL of ultrapure water (>18.2 MΩ·cm) containing 40 mg cetyltrimethylammonium bromide (CTAB) at room temperature. After 15 min of vigorous stirring, 3.0 mL of 2 M Zn(NO_3_)_2_·6H_3_O aqueous solution was added dropwise to the mixture. The resulting suspension was continuously stirred for 2 h, followed by centrifugation to collect the milky-white ZIF-L nanorods, which were subsequently washed several times with ultrapure water and N,N-dimethylformamide (DMF). The purified ZIF-L nanorods were then redispersed in 300 mL DMF, and 30 mg of Fe(NO_3_)_3_·9H_2_O was subsequently introduced under ultrasonication for homogeneous dispersion. The mixture was treated at 140 °C for 2 h to achieve topological transformation from ZIF-L into Fe@ZIF-8. Finally, the product was pyrolyzed at 1100 °C for 2 h under Ar gas flow with a heating rate of 10 °C/min, yielding Fe_SA_@NC.

**Synthesis of Fe_3_O_4_/Fe_SA_@NC nanorods**

To elucidate the formation mechanism and optimal conditions for Fe_3_O_4_ clusters, 30 mg of as-prepared Fe_SA_@NC was purged for 30 min with a N_2_/O_2_ mixed gas (20% O_2_), followed by immediate sealing in ampoules under controlled ambient pressures. The sealed ampoules used for the secondary pyrolysis were custom-made quartz ampoules with 15 mm in inner diameter and 70 mm in length. The total internal volume was approximately 8.2 mL. The oxygen-adsorbed Fe_SA_@NC samples were subsequently subjected to secondary pyrolysis in a tube furnace at 800 °C for 2 h (heating rate: 20 °C min^−1^) to drive cluster formation (denoted as Fe_3_O_4_/Fe_SA_@NC).

**Synthesis of Fe_SA_@NC-Ar nanorods**

Fe_SA_@NC powder was placed in tube furnace. The furnace was purged with high purity Ar for 30 min to remove residual air. Subsequently, the sample was heated to 800 °C for 2 h under Ar flow with a heating rate of 20 °C min^−1^. After cooling to room temperature under Ar protection, the resulting product was collected (denoted as Fe_SA_@NC-Ar).

**Characterizations**

The crystal structure of the samples was characterized by X-ray diffraction (XRD, Bruker D8 Advance, Cu Kα radiation λ = 1.5418 Å), while morphological features were examined using field-emission scanning electron microscopy (FE-SEM, ZEISS SUPRA™ 55) and high-resolution transmission electron microscopy (HR-TEM, JEOL JEM-3200FS). Atomic-scale imaging was performed via spherical aberration-corrected HAADF-STEM (FEI Titan Themis 60-300), with chemical composition analyzed by X-ray photoelectron spectroscopy (XPS, Thermo Fisher ESCALAB Xi+) and inductively coupled plasma optical emission spectroscopy (ICP-OES, Agilent 5100 VDV). The ^57^Fe Mössbauer spectra (MES) of the catalysts were measured on a MS963rd (Czech Republic, Iron Analytics s.r.o) spectrometer. Mössbauer measurements at 77K were performed in a helium free cryostat (Montana Cryoadvance 50). Surface vibrational modes were probed by Raman spectroscopy (Renishaw in Via, 532 nm laser), and porosity was evaluated through N_2_ adsorption-desorption measurements (Micromeritics/ASAP 2460, degassed at 150 °C for 12 h). Electronic properties were systematically investigated using ultraviolet photoelectron spectroscopy (UPS, Thermo Fisher ESCALAB 250Xi, He I 21.2 eV) for work function determination, electron paramagnetic resonance (EPR, CIQTEK EPR300, X-band 9.85 GHz) for unpaired electron quantification, and X-ray absorption fine structure (XAFS) spectroscopy at the Fe K-edge (Beijing Synchrotron Radiation Facility, BSRF 1W1B beamline, fluorescence mode).

**Electrochemical measurements**

The ORR performance of the prepared catalysts was evaluated using a standard three-electrode system on a CHI 760E electrochemical workstation (Shanghai Chenhua, China) in freshly prepared 0.1 M KOH electrolyte. The electrochemical cell consisted of a 3 mm diameter glassy carbon (GC) working electrode, a saturated calomel electrode (SCE) as reference electrode, and a high-purity carbon rod as counter electrode. To prepare the catalyst ink, 5 mg of catalyst was ultrasonically dispersed in a homogeneous mixture containing 480 μL ethanol and 20 μL Nafion solution (5 wt%). The well-dispersed ink was then drop-cast onto a mirror-polished GC electrode, maintaining a consistent catalyst loading of approximately 0.42 mg cm^−2^ for all samples including the Pt/C benchmark. Prior to electrochemical measurements, the electrolyte was saturated with oxygen by purging with high-purity O_2_ for at least 15 minutes. All measured potentials were converted to the reversible hydrogen electrode (RHE) scale using the following equation:

$$E_{RHE}=E_{SCE}+0.244+0.0591\times pH$$

The working electrode was activated by performing 30 cycles of cyclic voltammetry (CV) between 0.2–1.1 V vs. RHE at a scan rate of 20 mV s^-1^ to obtain stable linear sweep voltammetry (LSV) curves. Reported LSV curves were subsequently collected from 0.2-1.1 V vs. RHE at a scan rate of 1 mV s^−1^ with electrode rotation rates varying from 900 to 2025 rpm. Rotating ring-disk electrode (RRDE) measurements were conducted to quantitatively determine the hydrogen peroxide yield and electron transfer number (*n*) using the following equations:

$$H_{2}O_{2}(\%)=200\times(I_{R}/N)/(I_{R}/N+I_{D})$$

$$n=4\times I_{D}/(I_{R}/N+I_{D})$$

where *I*_R_ is the ring current, *I*_D_ is the disk current, and N represents the ring collection efficiency (N=0.37).

**Zn-air battery tests**

The ZAB was constructed using a polished 0.5-mm-thick zinc foil anode and an air cathode prepared by depositing the catalyst onto carbon cloth (loading: 0.5 mg cm^−2^ for Fe_3_O_4_/Fe_SA_@NC and Pt/C benchmarks). To enhance the charging capability of ZABs, 0.5 mg cm^-2^ of RuO_2_ was also deposited on the carbon cloth. The electrolyte consisted of an aqueous 6.0 M KOH solution containing 0.2 M Zn(Ac)_2_. Catalyst ink preparation followed the identical protocol to the electrocatalytic tests described earlier. Electrochemical measurements, including open-circuit voltage (OCV), discharge polarization curves, and power density plots, were recorded on a CHI 760E workstation. Rate capability and specific capacity were evaluated using a battery testing system (Land CT3001A) at ambient temperature. The rate performance was assessed through galvanostatic discharge at progressively increasing current densities (2, 5, 10, 20 mA cm^-2^) followed by stepwise return to 5 and 2 mA cm^-2^. Specific capacity was determined by constant-current discharge and normalized to the mass of consumed zinc. For rechargeable ZAB testing, the catalyst loading was increased to 1 mg cm^−2^. Long-term stability experiments were conducted under static air (ambient O_2_) at ~25°C, with cycling parameters matching practical operating conditions.

**Anion exchange membrane fuel cell (AEMFC) tests**

The membrane electrode assembly (MEA) was fabricated by spray-coating catalyst inks onto both sides of a PAP-TP-85 membrane (Versogen, 15 μm). The cathode catalyst layer was prepared using PiperION A5-HCO_3_ (5 wt.% in ethanol) as the ionomer. The ionomer/catalyst (I/C) ratio was optimized and determined to be 0.25. The cathode ink consisted of Fe_3_O_4_/Fe_SA_@NC or Fe_SA_@NC catalysts, while the anode employed commercial PtRu/C (40 wt% Pt, 20 wt% Ru), both homogenized with ionomer, isopropanol, and ultrapure water via ultrasonication. Precise catalyst loadings of 0.4 mg_PtRu_ cm^-2^ (anode) and 2.0 mg cm^-2^ (cathode catalyst) were maintained using calibrated manual spraying. Prior to assembly, the catalyst-coated membrane (CCM) was immersed in 1 M KOH overnight. Performance evaluation was conducted on a fuel cell test station (Scribner 850e) in voltage-scan mode (scan rate: 10 mV s^−1^). The cell operated at 80°C with fully humidified H_2_ (99.999%) and O_2_ (99.99%) supplied at 1 L min^−1^ (anode/cathode) under backpressure of absolute 200 kPa. Relative humidity (RH) was maintained at 100% via in-line humidifiers at matching temperatures.

**Computational details**

**First-principle calculations**

All first-principle calculations were performed using spin-polarized density-functional theory (DFT) methods as implemented in Vienna Ab initio Simulation Package (VASP 6.4)^[1]^. Generalized gradient approximation (GGA) with Perdew-Burke-Ernzerhof (PBE) functional was selected to describe the exchange-correlation interaction^[2]^. The ion-electron interaction was described by projector augmented-wave (PAW) method with a kinetic energy cutoff of 520 eV. Van de Waals (vdW) interactions were considered using the Grimme’s DFT-D3 method^[3]^. For sampling the Brillouin zone, Monkhorst-Pack grids of 2 × 2 × 1 and 5 × 5 × 1 k-point were used to optimize the model structures and investigate the electronic properties, respectively. The structural relaxations were carried out until the total energy and force were less than 10^-5^ eV per atom and 10^-2^ eV Å^-1^, respectively. The model Fe_SA_@NC and Fe_3_O_4_/Fe_SA_@NC were based on a 6 × 6 graphene with lattice constants of *a* = 14.77 Å and *b* = 14.77 Å. A sufficient vacuum slab of 20 Å along the *z* axis was added so that the interaction between the periodically repeated unit cells can be neglected. The VASPKIT was used to post-process the numerical results of VASP^[4]^.

The calculation of Gibbs free energy change (Δ*G*) for ORR was based on the computational hydrogen electrode (CHE) model ^[5]^, given by:

$\begin{aligned} \boldsymbol{\Delta}\boldsymbol{G}\boldsymbol{=}\boldsymbol{\Delta}\boldsymbol{E}\boldsymbol{+}\boldsymbol{\Delta}\boldsymbol{E}_{\mathbf{ZPE}}\boldsymbol{-}\boldsymbol{T}\boldsymbol{\Delta}\boldsymbol{S}\boldsymbol{+}\boldsymbol{eU}\boldsymbol{+}\boldsymbol{\Delta}\boldsymbol{G}_{\mathbf{pH}}\boldsymbol{\#(}\boldsymbol{1}\boldsymbol{)} \end{aligned}$where ∆*E* is the electronic energy difference between the free-standing and adsorption states of reaction intermediates; ∆*E*_ZPE_ and ∆*S* are the changes in zero-point energies and entropy, respectively, which can be obtained from the vibrational frequency calculations; *T* is the temperature, set to be 298.15 K in this work; *e* and *U* are the number of electrons transferred and the electrode potential applied, respectively. ∆*G*_pH_ is the free energy correction of pH, which can be obtained from ∆*G*_pH_ = *K*_B_*T* × pH × ln10. In this work, H_2_ and H_2_O were used as the reference states. Hence a set of equivalent reactions for the ORR mechanism were used to determine Δ*G*. The complete ORR catalytic process in alkaline condition includes the following four elementary steps:

O_2_(g) + H_2_O + e¯ → OOH^*^ + OH¯ (i)

OOH^*^ + e¯ → O^*^ + OH¯ (ii)

O^*^ + H_2_O + e¯ → OH^*^ + OH¯ (iii)

OH^*^ + e¯ → OH¯ (iv)

where * indicates the adsorption site and steps (i−iv) represent the four-electron transfer processes. The reaction free energy of (i-iv) for ORR can be calculated using the following equations:

$$\begin{aligned} \boldsymbol{\Delta}\boldsymbol{G}_{\boldsymbol{1}}\boldsymbol{=}\boldsymbol{\Delta}\boldsymbol{G}_{\mathbf{OOH}\mathbf{*}} \boldsymbol{-} \boldsymbol{4}\boldsymbol{.}\boldsymbol{92}\boldsymbol{\#}\left( \boldsymbol{2} \right) \end{aligned}$$

$$\begin{aligned} \boldsymbol{\Delta}\boldsymbol{G}_{\boldsymbol{2}}\boldsymbol{=}\boldsymbol{\Delta}\boldsymbol{G}_{\mathbf{O}\mathbf{*}} \boldsymbol{-} \boldsymbol{\Delta}\boldsymbol{G}_{\mathbf{OOH}\mathbf{*}}\boldsymbol{\#}\left( \boldsymbol{3} \right) \end{aligned}$$

$$\begin{aligned} \boldsymbol{\Delta}\boldsymbol{G}_{\boldsymbol{3}}\boldsymbol{=}\boldsymbol{\Delta}\boldsymbol{G}_{\mathbf{OH}\mathbf{*}} \boldsymbol{-} \boldsymbol{\Delta}\boldsymbol{G}_{\mathbf{O}\mathbf{*}}\boldsymbol{\#}\left( \boldsymbol{4} \right) \end{aligned}$$

$$\begin{aligned} \boldsymbol{\Delta}\boldsymbol{G}_{\mathbf{4}}\mathbf{=}\mathbf{-}\boldsymbol{\Delta}\boldsymbol{G}_{\mathbf{OH}\mathbf{*}} \boldsymbol{\#}\left( \mathbf{5} \right) \end{aligned}$$

**Constant-potential *ab initio* molecular dynamics calculations.**

The kinetics of hydrogenation of OO* was studied by using the *ab initio* molecular dynamics (AIMD) and Slow-Growth method as implemented in VASP 6.4. In AIMD simulations, explicit water solvation model (45 H_2_O molecules) combined with implicit solvation model were used. The implicit solvation model was based on the VASPsol^[6,7]^ with the relative permittivity of 80. The linearized Poisson−Boltzmann model with a Debye length of 3.0 Å was used to mimic the compensating charge. The applied potential with respect to SHE (standard hydrogen electrode) was obtained by:

$U_{q}\left( V/\mathrm{SHE} \right)=-4.6-{\phi_{q}}/e$	(6)

where *ϕ*_q_ is the work function of the charged surface in aqueous solution and 4.6 V is the work function of the H_2_/H^+^ couple at standard conditions. In addition, to evaluate the influence of pH value in the experiment, the relationship between *U*_RHE_ (Reversible hydrogen electrode) and *U*_SHE_ is listed as follows:

$U_{\mathrm{RHE}}=U_{\mathrm{SHE}}+{k_{B}T\ln\left( 10 \right)\mathrm{pH}}/e$	(8)

The cutoff energy of the plane-wave basis was 400 eV. PBE functional with D3 van der Waals correction was employed. Time step in AIMD was set to be 1 femtosecond, and the collective variable (CV) increment was set to be 0.0005 to 0.0008 Å to make sure the convergence. A Nosé-Hoover thermostat was adopted to keep temperature constant at 300 K. Gamma point of Brillouin zone was used. The convergence criteria for the electronic step was set to be 10^-4^ eV, and POMASS of H was set to be 3.

[1] G. Kresse,J. Hafner, *Phys. Rev. B.* **1993**, *47*, 558-561.

[2] J. P. Perdew, K. Burke,M. Ernzerhof, *Phys. Rev. Lett.* **1996**, *77*, 3865-3868.

[3] S. Grimme, S. Ehrlich,L. Goerigk, *J. Comput. Chem.* **2011**, *32*, 1456-1465.

[4] V. Wang, N. Xu, J. C. Liu, G. Tang,W. T. Geng, *Comput. Phys. Commun.* **2021**, *267*, 108033.

[5] A. A. Peterson, F. Abild-Pedersen, F. Studt, J. Rossmeisl,J. K. Nørskov, *Energy Environ. Sci.* **2010**, *3*, 1311-1315.

[6] K. Mathew, V. S. C. Kolluru, S. Mula, S. N. Steinmann,R. G. Hennig, *J. Chem. Phys.* **2019**, *151*, 234101.

[7] K. Mathew, R. Sundararaman, K. Letchworth-Weaver, T. A. Arias,R. G. Hennig, *J. Chem. Phys.* **2014**, *140*, 084106.

**Figure S1**. Schematic illustration of the morphology and atomic structure of ZIF-8 nanorods.

**Figure S2**. SEM images of (a, b) ZIF-L and (c, d) Fe@ZIF-8 nanorods.

**Figure S3**. XRD patterns of Fe@ZIF-8 nanorods.

**Figure S4**. (a) XRD pattern, (b) SEM image, (c) TEM image, and (d) EDS mapping images of Fe_3_O_4_/Fe_SA_@NC nanorods.

**Figure S5**. (a) AC-HAADF-STEM image of Fe_SA_@NC. (b) Pseudocolor HAADF-STEM image of single-atom distribution based on Fe_SA_@NC.

**Figure S6**. Sizes of ampoules used for the secondary pyrolysis.

**Figure S7**. (a) AC-HAADF-STEM image of Fe_3_O_4_/Fe_SA_@NC. (b) Pseudocolor HAADF-STEM image of single-atom/cluster distribution based on Fe_3_O_4_/Fe_SA_@NC.

**Figure S8**. (a) Low-magnification HAADF-STEM image of Fe_3_O_4_/Fe_SA_@NC. As can be seen, the bright particles belonging to Fe_3_O_4_ clusters are uniformly distributed across the carbon matrix. (b) single-atom (yellow circles)/cluster distribution based on HAADF-STEM of Fe_3_O_4_/Fe_SA_@NC. (c) EDS spectrum of Fe_3_O_4_/Fe_SA_@NC.

**Figure S9**. HRTEM images and corresponding FFT patterns of Fe_3_O_4_/Fe_SA_@NC.

**Figure S10**. (a) Low-magnified TEM image of Fe_3_O_4_/Fe_SA_@NC-1(secondary pyrolysis at 5×10^−4^ mbar). (b) HRTEM images of Fe_3_O_4_/Fe_SA_@NC-1. (c) Lattice fringes of Fe_3_O_4_ crystallite.

**Figure S11**. (a) Low-magnified TEM image of Fe_3_O_4_/Fe_SA_@NC-2 (secondary pyrolysis at 10^−4^ mbar). (b) HRTEM image of Fe_3_O_4_/Fe_SA_@NC-2. (c-e) EDS mapping images of Fe and O.

**Figure S12**. (a) Hysteresis loops and (b) the enlarged detail of Fe_3_O_4_/Fe_SA_@NC and Fe_SA_@NC.

**Figure S13**. (a) N_2_ adsorption/desorption isotherms and (b) corresponding pore size distributions of Fe_3_O_4_/Fe_SA_@NC and Fe_SA_@NC.

**Figure S14**. XPS survey spectra of (a) Fe_3_O_4_/Fe_SA_@NC and (b) Fe_SA_@NC.

**Figure S15**. High-resolution C 1s XPS spectrum of (a) Fe_3_O_4_/Fe_SA_@NC and (b) Fe_SA_@NC.

**Figure S16**. Fe 2p XPS spectra of Fe_3_O_4_/Fe_SA_@NC and Fe_SA_@NC.

**Figure S17**. Zn 2p XPS spectra of (a) Fe_3_O_4_/Fe_SA_@NC and (b) Fe_SA_@NC.

**Figure S18**. UPS spectra of Fe_3_O_4_/Fe_SA_@NC and Fe_SA_@NC.

**Figure S19**. Fe K-edge EXAFS fittings curve of Fe_3_O_4_/Fe_SA_@NC at *k* space.

s

**Figure S20**. EXAFS *R* space fitting curve of Fe_3_O_4_/Fe_SA_@NC in the range of 1-2 Å.

**Figure S21**. EXAFS *R* space fitting curve of Fe_SA_@NC.

**Figure S22**. (a) LSV curve and (b) Tafel slope of NC in 0.1 M KOH.

**Figure S23**. TOF and *J*_k_ of relevant catalysts at (a) 0.85 V vs RHE and (b) 0.9 V vs RHE.

**Figure S24**. (a) LSV curves and (b) corresponding *E*_1/2_ of Fe_3_O_4_/Fe_SA_@NC obtained in different secondary pyrolysis temperature.

**Figure S25**. (a) LSV curves and (b) corresponding *E*_1/2_ of Fe_3_O_4_/Fe_SA_@NC with different Fe precursor dosage.

**Figure S26**. (a) LSV curves and (b) corresponding *E*_1/2_ of Fe_3_O_4_/Fe_SA_@NC obtained in different ambient pressures.

**Figure S27**. (a) LSV curves and (b) corresponding K-L plots of Fe_3_O_4_/Fe_SA_@NC recorded at different rotation speed ranging from 900 to 2500 rpm.

**Figure S28**. Methanol tolerance test of Fe_3_O_4_/Fe_SA_@NC and commercial Pt/C.

**Figure S29**. OCV curve of Fe_3_O_4_/Fe_SA_@NC and commercial Pt/C based ZABs.

**Figure S30**. Specific discharging capacities at 20 mA cm^−2^ of Fe_3_O_4_/Fe_SA_@NC and commercial Pt/C based ZABs.

**Figure S31**. The accelerated stress test (AST) protocol procedure.

**Figure S32**. AST performance at begin-of-life and end-of-life of Fe_SA_@NC.

**Figure S33**. (a) Voltage retention obtained by using Fe_3_O_4_/Fe_SA_@NC as cathode catalyst during AEMFC stability tests. (b) High-Frequency Resistance by using Fe_3_O_4_/Fe_SA_@NC as cathode catalyst during AEMFC stability tests.

**Figure S34**. Comparison of peak power density of Fe-based metal catalysts.

**Figure S35**. *In-situ* ATR-SEIRAS of (a) Fe_3_O_4_/Fe_SA_@NC, (b) Fe_SA_@NC and (c) Pt/C catalyst.

**Figure S36**. *In-situ* FTIR spectra of OOH* and HOOH* on (a) Fe_3_O_4_/Fe_SA_@NC and (b) Fe_SA_@NC catalysts. (c) The detailed variations of OOH* and HOOH* with potentials.

**Figure S37**. (a) LSV curves measured in 0.1M KOH based D_2_O and H_2_O and (b) corresponding Tafel slopes of Fe_3_O_4_/Fe_SA_@NC and Fe_SA_@NC catalysts.

**Figure S38**. (a) *In situ* ATR-SEIRAS of H-O-H on Fe_3_O_4_/Fe_SA_@NC and Fe_SA_@NC catalysts. (b) *In-situ* ATR-SEIRAS of *OH on Fe_3_O_4_/Fe_SA_@NC and Fe_SA_@NC catalysts.

**Figure S39**. ORR mechanism of water dissociation for Fe_3_O_4_/Fe_SA_@NC and Fe_SA_@NC.

In detail, O_2_ preferentially binds to Fe-N_4_ sites to form Fe-N_4_-O_2_ adducts, and its subsequent conversion is facilitated by interfacial hydrogen bonding with nearby water molecules. Concurrently, Fe_3_O_4_ clusters as hydrolysis sites can continuously supply protons for Fe-N_4_-O_2_, thus accelerating the PCET process of ORR. A surface proton from Fe_3_O_4_-OH_2_ can then transfer to the adjacent Fe-N_4_-O_2_, yielding Fe_3_O_4_-OH and Fe-N_4_-OOH, followed by further reduction to Fe-N_4_=O and OH^−^. Finally, Fe-N_4_=O undergoes subsequent PCET steps to regenerate the initial state.

**Figure S40**. The optimized structures of Fe_SA_@NC and the adsorption of OOH*, O* and OH* (The orange, brown, blue, red and white balls indicate the Fe, C, N, O and H, respectively).

**Figure S41**. The optimized structures of Fe_3_O_4_/Fe_SA_@NC and the adsorption of OOH*, O* and OH* (The orange, brown, blue, red and white balls indicate the Fe, C, N, O and H, respectively).

**Figure S42.** Optimized structure of a) Fe_SA_@NC and b) Fe_3_O_4_/Fe_SA_@NC catalysts. c) The COHP analysis for Fe-N of Fe_SA_@NC and Fe_3_O_4_/Fe_SA_@NC. d) The PDOS for Fe_SA_@NC and Fe_3_O_4_/Fe_SA_@NC, the d-band centers are labelled.

**Table S1**. Elemental analysis result of the prepared catalysts.

| Catalysts | Fe content (wt.%) | Zn content (wt.%) |
| --- | --- | --- |
| Fe_3_O_4_/Fe_SA_@NC | 0.81 ± 0.02 | 0.26 ± 0.01 |
| Fe_SA_@NC | 0.82 ± 0.03 | 0.29 ± 0.03 |

The element contents were determined by ICP-OES analysis.

T**able S2**. Summary of synthesis parameters for each sample.

| Sample | Precursor | Primary pyrolysis | Secondary pyrolysis atmosphere/pressure | Secondary pyrolysis procedure |
| --- | --- | --- | --- | --- |
| Fe_SA_@NC | Fe@ZIF-8 | 900°C/ 2h/Ar | **−** | **−** |
| Fe_SA_@NC-Ar | Fe_SA_@NC | **−** | Ar/Atmosphere | 800°C/2h |
| Fe_3_O_4_/Fe_SA_@NC | Fe_SA_@NC | **−** | N_2_/O_2_ (80:20)/  10^−5^ mbar | 800°C/2h |
| Fe_3_O_4_/Fe_SA_@NC-1 | Fe_SA_@NC | **−** | N_2_/O_2_ (80:20)/  5×10^−5^ mbar | 800°C/2h |
| Fe_3_O_4_/Fe_SA_@NC-2 | Fe_SA_@NC | **−** | N_2_/O_2_ (80:20)/  10^−4^ mbar | 800°C/2h |

The secondary pyrolysis was performed in sealed quartz ampoules (inner diameter: 15 mm, inner length: 7 mm, total internal volume: 8.2 mL). The pressure control was achieved as follows: i) The ampoule containing Fe_SA_@NC powder was evacuated using a turbomolecular pump system to a base pressure of 10^−5^ mbar. ii) The ampoule was then backfilled with a N_2_/O_2_ mixture (20% O_2_) to atmospheric pressure. iii) The evacuation-backfill cycle was repeated three times to ensure complete removal of residual air. iv) The ampoule was finally evacuated to the target vacuum level (10^−5^ mbar, 5×10^−5^ mbar, or 10^−4^ mbar) before being sealed with a hydrogen-oxygen torch.

**Table S3**. The contents of different Fe species in Fe_3_O_4_/Fe_SA_@NC.

| Fe species | Area ratio (%) | δ (mm s^−1^) | H (T) |
| --- | --- | --- | --- |
| Fe-N-C | 63.35 | 0.48 | − |
| Fe_3_O_4_ A | 13.53 | 0.31 | 50.4 |
| Fe_3_O_4_ B (Fe^3+^) | 15.11 | 0.40 | 52.5 |
| Fe_3_O_4_ B (Fe^2+^) | 8.01 | 0.88 | 48.3 |

The contents of Fe species were determined by ^57^Fe Mössbauer spectra at 77 K.

**Table S4**. The content of different N types for the prepared catalysts.

| Catalysts | Pyridinic-N | Oxidized-N | Graphitic-N | Fe-N |
| --- | --- | --- | --- | --- |
| Fe_3_O_4_/Fe_SA_@NC | 37.2 | 16.4 | 30.7 | 15.7 |
| Fe_SA_@NC | 34.1 | 10.8 | 34.4 | 20.7 |

**Table S5.** Fitting parameters of Fe K-edge EXAFS curve of Fe_3_O_4_/Fe_SA_@NC.

| **Fe_3_O_4_/Fe_SA_@NC** | **CN** | **R (Å)** | **σ^2^ (10^-3^ Å^2^ )** | **Δ*E*_0_ (eV)** | **R factor (%)** |
| --- | --- | --- | --- | --- | --- |
| Fe-N | 1.4±0.14 | 2.03 | 3.0±0.42 | 2.248±0.22 | 0.38 |
| Fe-O | 0.629±0.09 | 2.038 | 1.0±0.18 | 2.248±0.22 |  |
| Fe-Fe | 0.6±0.04 | 2.384 | 7.81±0.25 | 2.248±0.22 |  |
| Fe-O-Fe | 0.532±0.21 | 2.822 | 9.21±2.12 | 2.248±0.22 |  |

**Table S6.** Fitting parameters of Fe K-edge EXAFS curve of Fe_3_O_4_/Fe_SA_@NC.

| **Fe_3_O_4_/Fe_SA_@NC** | **CN** | **R (Å)** | **σ^2^ (10^-3^ Å^2^ )** | **Δ*E*_0_ (eV)** | **R factor (%)** |
| --- | --- | --- | --- | --- | --- |
| Fe-N* | 2.93 | 1.99 | 3.72 | -5.10 | 0.21 |
| Fe-O* | 0.81 | 2.26 | 8.58 | -5.10 |  |

*The fitting range in Fe K-edge EXAFS curve of Fe_3_O_4_/Fe_SA_@NC is set at 1-2 Å, in which the contribution of first-shell Fe-Fe and second-shell Fe-O-Fe was deducted. As can be seen, the CN of Fe-N in Fe_3_O_4_/Fe_SA_@NC has increased to 2.93. After deducting the contribution of Fe-O coordination in Fe_3_O_4_ clusters, the CN of Fe-N in Fe_SA_@NC substrate is close to 4. Thus, it is reasonable to descript Fe_3_O_4_/Fe_SA_@NC as Fe_3_O_4_ and Fe-N_4_ coupled structure.

**Table S7**. Fitting parameters of Fe K-edge EXAFS curve of Fe_SA_@NC.

| **Fe_SA_@NC** | **CN** | **R (Å)** | **σ^2^ (10^-3^ Å^2^ )** | **Δ*E*_0_ (eV)** | **R factor (%)** |
| --- | --- | --- | --- | --- | --- |
| Fe-N | 3.83±0.13 | 1.96 | 9.42±0.29 | -4.63±0.38 | 1.1 |

**Table S8**. Summary of ORR performance of Fe_3_O_4_/Fe_SA_@NC and reported Fe-N-C catalysts with different modifications in alkaline conditions.

| Catalysts | *E*_onset_ (V *vs.* RHE) | *E*_1/2_ (V *vs.* RHE) | ref |
| --- | --- | --- | --- |
| **Fe_3_O_4_/Fe_SA_@NC** | **1.05** | **0.953** | **This work** |
| FeN_4_-Cl_1_^hs^ | 1.02 | 0.917 | Adv. Mater.  2024, 36, 2404692. |
| FeN_4_@C-O-C | 1.08 | 0.92 | Angew. Chem. Int. Ed.  2023, 62, e202308349. |
| FeN_4_S_1_-P_1_2nd | 1 | 0.912 | Energy. Environ. Sci.  2024, 17, 249-259. |
| FeN_4_@P-O | 1.01 | 0.912 | ACS. Energy. Lett  2023, 8, 4531-4539. |
| FeN_4_@oxidized S | 1.01 | 0.91 | Adv. Funct. Mater.  2023, 33, 2304074. |
| FeN_4_@Ti_3_N_2_Ac | 1 | 0.92 | J. Mater. Chem. A.  2022, 10, 24041-24050. |
| FeN_4_-NV^1^ | 0.99 | 0.902 | J. Am. Chem. Soc.  2024, 146, 4803-4813. |
| FeN_4_-P_2_2nd | 1.01 | 0.9 | J. Am. Chem. Soc.  2023, 145, 3647-3655. |
| FeN_4_@Fe_7_Ac^c^ | 1.02 | 0.894 | Adv. Funct. Mater.  2024, 34, 2315150. |
| FeN_4_S_1_^hs b^ | 1.05 | 0.887 | Adv. Mater.  2018, 30, 1804504. |
| Fe-N_4_/CNCl | 1.02 | 0.917 | Adv. Mater.  2024, 36, 2404692. |
| FeMn-N-C | 1.05 | 0.92 | Adv. Mater.  2024, 36, 2405763. |
| SA-Fe-NHPC | 1.01 | 0.93 | Adv. Mater.  2020, 32, 1907399. |
| FeN_4_-O-NCR | 1.05 | 0.942 | Adv. Mater.  2022, 34, 2202544. |

**Table S9**. Summary of H_2_-O_2_ AEMFCs performance of Fe_3_O_4_/Fe_SA_@NC and reported non-noble metal in alkaline conditions.

| Catalysts | Cathode loading (mg cm^−2^) | Anode loading (mg cm^−2^) | Peak power density (W cm^−2^) | References |
| --- | --- | --- | --- | --- |
| **Fe_3_O_4_/Fe_SA_@NC** | **2.0** | **PtRu, 0.4** | **0.909** | **This work** |
| N-C-CoO_x_ | 2.4 | PtRu, 0.1 | 0.73 | Angew. Chem. Int. Ed.  2019, 58, 1046-1051. |
| N-doped Carbon | 1.0 | PtRu, 0.6 | 0.7 | J. Power. Sources.  2017, 372, 82-90. |
| meso-Fe-N-C | 4.0 | PtRu, 0.4 | 0.584 | ACS. Catal.  2021, 11, 74-81. |
| Ce/Fe-NCNW | 1.0 | PtRu, 0.75 | 0.496 | ACS. Catal.  2020, 10, 2452-2458. |
| Fe/N/C | 4.0 | PtRu, 0.4 | 0.473 | ACS. Catal.  2017, 7, 6485-6492. |
| Fe/N/C | 2.0 | PtRu, 0.4 | 0.45 | ACS. Catal.  2017, 7, 6485-6492. |
| Fe-N/C | 2.0 | Pt, 0.5 | 0.38 | J. Am. Chem. Soc.  2016, 138, 15046-15056. |
| Pt/C | 0.5 | Pt, 0.5 | 0.818 | ACS. Macro. Lett.  2017, 6, 1089-1093. |
| FeCo-NHC | 1.0 | PtRu,0.2 | 0.569 | Nat. Commun.  2024, 14, 1882 |
| N-graphite | 0.7 | PtRu,0.7 | 0.361 | Appl. Catal. B Environ  2024, 357, 124319 |
| Cu-N-C/GC | 2.5 | PtRu, 0.4 | 0.324 | Angew. Chem. Int. Ed.  2022, 61, e202211098 |
| Fe_3_O_4_/La_2_O_3_@N,O-CNSs | 1.0 | Pt, 0.1 | 0.1487 | Adv. Funct. Mater.  2024, 34, 2309886. |
| FeMn-N-MPC | 2.0 | PtRu,0.8 | 0.474 | Appl. Catal. B Environ.  2022, 306, 121113. |
| *g*-CAN-Pc | 2.0 | Pt, 0.2 | 0.616 | Angew. Chem. Int. Ed.  2025, e16530 |

**Table S10**. The population analysis for Fe_SA_@NC and Fe_3_O_4_/Fe_SA_@NC based on Löwdin method.

| Atom | Fe_SA_@NC | Fe_3_O_4_/Fe_SA_@NC |
| --- | --- | --- |
| Fe | 1.05 e | 1.03 e |
| N1 | -0.42 e | -0.45 e |
| N2 | -0.42 e | -0.43 e |
| N3 | -0.42 e | -0.42 e |
| N3 | -0.42 e | -0.42 e |
